# Transcranial Photobiomodulation Modulates Oxidative Stress Biomarkers and Complex IV Activity in Anhedonic-Like Behavior

**DOI:** 10.1007/s11064-026-04681-2

**Published:** 2026-02-21

**Authors:** Luciana Bortoluzzi, Rafael Colombo, Karoline Borges da Motta Pinto, Lucas Henriques Viscardi, Ricardo Missiaggia, Douglas Jean Turella, Lisandra Schwantess, Mirian Salvador, Catia Santos Branco, Marina Rigotti, Ellen Scotton, Tainá Schons, Silene Bazi Ribeiro, Marco Antonio Caldieraro, Adriane R. Rosa

**Affiliations:** 1 Postgraduate Program in Biological Sciences, Pharmacology and Therapeutics - Institute of Basic Health Sciences, Federal University of Rio Grande do Sul, Porto Alegre, Rio Grande do Sul Brazil; 2 University of Caxias do Sul, Caxias do Sul, Rio Grande do Sul Brazil; 3 Laboratory of Molecular Psychiatry, Hospital Clinic of Porto Alegre, Porto Alegre, Rio Grande do Sul Brazil; 4 Postgraduate Program in Psychiatry and Behavioral Sciences, Faculty of Medicine, Federal University of Grande do Sul, Porto Alegre, Rio Grande do Sul Brazil; 5 Experimental Research Center (CPE) and Clinical Research Center (CPC), Hospital de Clínicas de Porto Alegre (HCPA), Rua Ramiro Barcelos, 2350, Santa Cecília, Porto Alegre, Rio Grande do Sul 90410-000 Brazil; 6 Laboratory of Oxidative Stress and Antioxidants, Institute of Biotechnology, University of Caxias do Sul, Caxias do Sul, Rio Grande do Sul Brazil; 7 Caxias do Sul, Brazil

**Keywords:** Major depressive disorder (MDD), Treatment-resistant depression (TRD), Photobiomodulation (PBM), Transcranial photobiomodulation (TPBM), Photobiomodulation therapy (PBMT), Infrared (IR), Chronic mild stress (CMS)

## Abstract

Major depressive disorder (MDD) is a prevalent and complex condition with limited treatment success in many patients. Photobiomodulation (PBM), particularly transcranial PBM (tPBM) using red to near-infrared light, has emerged as a promising non-invasive intervention. However, optimal parameters and precise mechanisms remain unclear. This research aimed to analyze the effects of transcranial photobiomodulation (red and infrared) on behavioral and biological parameters related to MDD in a chronic mild stress (CMS) model. Male Wistar rats were exposed to CMS for five weeks and subsequently categorized into two groups—resilient (CMS-R) and susceptible (CMS-S)—based on their performance in the sucrose consumption test (SCT). The CMS-S group was further divided into three subgroups: (1) sham treatment, (2) tPBM red (600 nm), and (3) tPBM infrared (840 nm). A control group of non-stressed animals was included for baseline comparisons. Biological measures included lipid damage (TBARS), antioxidant defense (TEAC), mitochondrial complex IV activity (CCO), and nitric oxide (NO) concentration in the prefrontal cortex and blood were measured. As expected, post-tPBM treatment (both red and infrared groups) exhibited increased sucrose consumption compared to the sham (*p* < 0.001). The red and infrared presented higher serum TEAC levels than the sham and control groups, but these effects did not reach statistical significance (*p* = 0.306). In contrast, the red group showed lower peripheral TBARS levels than the sham group (*p* = 0.0048); such effect was similar to the control non-stress group. The infrared group showed higher NO levels within the hippocampus than the sham group *p* = 0.0134) and higher prefrontal CCO activity levels than the red group (*p* = 0.012), which was similar to the control non-stress group. Our study demonstrated that animals treated with tPBM using red (600 nm) or infrared (840 nm) wavelengths exhibited significant improvements in both behavioral and biological parameters in the CMS model. In particular, tPBM may offer therapeutic benefits by ameliorating oxidative stress and enhancing mitochondrial function, thereby presenting a promising alternative for the management of MDD.

## Introduction

Major depressive disorder (MDD) is a complex and multifaceted mental illness that presents with ongoing feelings of sadness, lack of interest in previously enjoyed activities, repetitive thoughts of death, and cognitive and physical symptoms [1]. According to the Global Burden of Disease Collaborative Network, MDD impacts 185 million individuals worldwide [2]. People with MDD often present impairment in occupational, familial, and social domains, concomitantly diminishing the overall quality of life [3].

MDD is often accompanied by physical comorbidities, which may contribute to a decreased life expectancy in individuals with MDD [4]. Indeed, depressive symptomatology carries consequences such as an increased incidence of cardiovascular diseases [5], including coronary artery disease, heart failure, and stroke [6]. Further, MDD increases the risk of suicidal ideation, attempts, and death, contributing to its disease burden [7].

The standard treatment recommendations typically encompass a combination of antidepressant pharmacotherapy and psychotherapeutic interventions [2]. However, it is noteworthy that a substantial proportion, approximately one-third of individuals diagnosed with MDD, fail to attain a satisfactory clinical response despite undergoing multiple therapeutic interventions [8]. Consequently, such cases are classified as treatment-resistant depression (TRD) [9]. Consequently, the importance of identifying and implementing effective adjuvant therapeutic interventions in managing TRD becomes patently evident.

Chronic Mild Stress (CMS), first described almost four decades ago, has become a popular model in developing drugs for treating MDD. This model has engendered a substantial number of scientific investigations, with nearly 1700 studies solely focusing on rat-based research [10]. Chronic stress exerts many harmful effects, encompassing mood, cognitive, and memory impairments. Research has shown that unpredictable stress can seriously affect the brain, including atrophy in the cortical and limbic regions, sensitization of the serotonergic system, increased levels of inflammatory proteins in the hippocampus, proliferation, and microglial activation, and disruptions in the HPA axis [11]. Moreover, this model has been extensively used to mimic animal models of depression.

Current research on mood disorders has focused heavily on the intricate relationship between systemic inflammation, oxidative stress, and the brain. The disparity between the production of reactive oxygen species (ROS) and the body’s capacity to detoxify them, commonly referred to as oxidative stress, has been associated with the emergence of MDD. This disparity can damage neuronal structures and neurotransmitter systems, which can contribute to the onset and persistence of depression [12]. Emerging evidence suggests that oxidative stress and systemic inflammation may synergistically exacerbate depressive symptoms, highlighting the intricate web of factors involved in the pathogenesis of MDD and offering potential targets for innovative therapeutic interventions [13].

Photobiomodulation (PBM) emerged as a promising treatment for MDD [14]. This therapy uses red to near-infrared (NIR) spectrum photons (600–1100 nm) to irradiate tissue [15]; using lasers or light-emitting diodes (LEDs) as the light source. The transcranial photobiomodulation modality (tPBM) is a novel therapy that employs visible or non-visible light delivered to the skull aiming to modulate the activity of the subjacent cortex. Studies have demonstrated that red and near-infrared light with wavelengths ranging from 630 to 810 nm can penetrate the skull at a rate of 0.2% to 10% [15]. Light in the red and NIR spectrum is absorbed by the cytochrome C oxidase (CCO), triggering a series of cellular and physiological events, such as stimulating mitochondrial metabolism, increasing adenosine triphosphate (ATP) production, decreasing oxidative stress, neuroinflammation, and neuronal apoptosis, repairing damaged hypoxic cells, modulation of target tissue function and augmented cerebral metabolism and perfusion by releasing nitric oxide (NO) [16, 17].

Additionally, tPBM has been utilized to treat mood disorders in both animal models and humans and has displayed the potential to alter the neuroinflammatory profile in the brains of young and old rats [17, 18]. Furthermore, tPBM is considered safe when delivered by trained professionals or self-administered at home, depending on the device used for the treatment [16]. It typically does not cause pain, significant heat, or tissue damage, making it a non-invasive and low-risk therapy [19].

Although t-PBM is a promising treatment for MDD, the ideal stimulation parameters, including the most effective wavelengths, are not yet established. Also, the mechanisms of its antidepressant effect are not completely understood. Therefore, this study aimed to analyze the impact of tPBM with two different wavelengths (red 600 nm and NIR 840 nm) on behavioral, peripheral, and central biological parameters related to the pathophysiology of MDD in a CMS model.

## Materials and Methods

### Animals

We used eighty-eight (*N* = 88) male Wistar rats from the Laboratory Animal Reproduction and Experimentation Center of the Federal University of Rio Grande do Sul (CREAL-UFRGS). The animals were two months old and weighed approximately 250–300 g. To calculate the sample size, we used the mean and standard deviation data for the sucrose consumption outcome of the control groups and those submitted to the CMS protocol, evaluated in the study by Wang and collaborators [20]. We used the ClinCalc system, available at (https://clincalc.com/Stats/SampleSize.aspx) for the calculation. We considered the difference between the means and standard deviations for each calculated outcome, an alpha error probability of 0.05 (*p* < 0.05), and a confidence interval of 90%. The estimated value was ten animals per group. However, based on the literature, it was considered that only 50% of animals subjected to stress would develop depressive-like behavior [21, 22]. Thus, to apply the CMS protocol and categorize groups as susceptible or resilient, a duplicated initial “N” is required. Therefore, 88 animals were needed (*n* = 4 in the pilot study; *n* = 14 animals excluded due to variation in sucrose consumption; *n* = 10 in the control group; *n* = 60 in the CMS group).

The animals were housed in the Physiology and Pharmacology Laboratory of the University of Caxias do Sul (UCS) under standard conditions of a 12-hour light/dark cycle, temperature of 22 ± 2 °C, relative air humidity of 40–60%, with standard food and water *ad libitum.* Before beginning the CMS protocol, the animals were quarantined and acclimated for 14 days, with three animals per box. After that, basal sucrose consumption was measured. During the CMS protocol, animals were housed individually and kept isolated until the experiments ended. The animals in the control group were housed in groups (2 per box) to prevent them from suffering isolation stress. This study was approved by the Animal Ethics Committee (CEUA) of the University of Caxias do Sul under number #002/2020 on 03/13/2020.

### Analysis of Light Penetration Energy Delivery

Before starting the experiment, we evaluated the delivery of light energy and the penetration of these lights into the tissues, in addition to heating on the skin surface using red (600 nm) and infrared (840 nm) LED lights. Tissue samples were used from four animals (two animals aged two months and two animals aged four months) of the same species as those subjected to the study protocol. The samples comprised the frontal portion of the head (skull), skin, and hair. The samples were irradiated, two with each wavelength, at the same point on the skull where the animals received the treatment. The amount of energy that penetrated the samples was measured for each of the wavelengths: the bone part with scalp and hair and the bone part with scalp and without hair. Therefore, no significant heating of the skin surface was observed. The bone part with scalp and without hair had better light penetration, with 17%±6% of red light (600 nm) and 19%±4% of infrared light (840 nm) applied to the skin surface able to cross these structures when compared to the bony part with scalp and hair (9%±3% of red light and 10%±2% of infrared light. Table 1 describes the doses used in this study. The samples were measured using an optical sensor connected to a digital source (Icel Manaus - model PS-6000). The samples were measured using an optical sensor connected to a digital source (Icel Manaus - model PS-6000). The surface temperature of the sample was detected by a thermographic camera (FLIR System AB). A digital caliper (MTX-316119) was used to measure the thickness of the irradiated bone region. The LED on the sensor was held in position for approximately 10 s, or until the penetration power readings stagnated at a maximum of 20 s, to obtain penetration readings (mW). This procedure was carried out in duplicate for each evaluation by the engineering sector of the company Tonederm^®^, which also provided the LED prototypes for application in this experiment. Despite the preliminary nature of the results, we acknowledge that a larger sample size is essential to substantiate these findings, and these findings should be replicated in further studies.

**Table 1:** Parameters used in transcranial photobiomodulation treatment

Parameter	Value	Value
Wavelength	600 nm	840 nm
Light source	LED	LED
Wave mode	continuous	continuous
Illumination time	150 s	150 s
Spot size	0,25 cm^2^	0,25 cm^2^
Total power output	400 mW	400 mW
Total output power density	1,6 W/cm^2^	1,6 W/cm^2^
Average light penetration in the PFC	17% ±6%	19% ± 4%
Estimated total delivery power in the PFC	68 mW ± 4,0	76 mW ± 3,0%
Estimated power density in the PFC	272 mW/cm^2^ ± 16,3	304 mW/cm^2^ ± 12,1
Total energy per session	10,2 J ± 0,6	11,4 J ± 0,4
Estimated fluency delivered to the PFC	40 J/cm^2^ ± 2,4	45 J/cm^2^ ± 1,8

### Sucrose Consumption-Baseline

After two weeks of quarantine and acclimatization, the rats were introduced to sucrose consumption. The animals were trained to consume a 1% sucrose solution twice a week for 30 days. After 14 h of food and water deprivation, the animals (now individualized) received a freshly prepared 1% sucrose solution for 60 min in a 100 ml bottle numbered according to each animal. Sucrose intake was calculated by weighing the bottles before and after the test. The basal sucrose consumption of each animal was evaluated in the last week.

Papp [23] found that animals showed variation in sucrose consumption before starting the stress protocol [24]. This significant variation may affect our results, as the sucrose test is the primary outcome in characterizing anhedonic behavior. This study demonstrated that 20% of the animals present either a high consumption, a reduced consumption, or a significant variation in sucrose consumption. Animals that fit this profile were excluded from the experiment (*n* = 14).

To divide the animals into stressed and control groups, they were paired based on their average sucrose consumption. Sucrose consumption was monitored weekly under similar conditions throughout the CMS protocol until the end of the study. After the test, food and water were replaced after a one-hour wait.

### Chronic Mild Stress Protocol (CMS)

The CMS protocol lasted ten weeks. Animals in the CMS group were exposed to stressors according to the method described by Papp et al. [25] and Willner et al. [26]. The rats belonging to the CMS group were placed in standard plastic boxes and kept separate from each other. They were exposed to the following stressors: two (02) weekly periods of food and water deprivation; two (02) weekly periods with the cage tilted at 45º; two (02) weekly periods of intermittent lighting on/off every two (02) hours; two (02) weekly periods with a dirty cage; two (02) weekly periods with the presence of an intruding animal; two (02) weekly periods with low-intensity stroboscopic lighting; two (02) weekly stress-free periods. For ten consecutive weeks, stress was induced once or twice daily (10–14 h for each stressor).

After conducting the sucrose consumption test in the fifth week of the CMS protocol, animals were divided into susceptible and resilient subgroups. The susceptible subgroup (CMS-S) consisted of animals that exhibited depressive-like behavior after the behavioral protocols. In contrast, the resilient subgroup (CMS-R) included animals that did not display such behavior. The definition of susceptible or resilient animals was guided by the outcome observed in the sucrose consumption test, defining susceptible animals as those with sucrose consumption lower than 40% of the baseline assessment value [23].

After 5 weeks of CMS, 50% of the animals were classified as susceptible to stress; treatment with tPBM red (600 nm) or tPBM infrared (840 nm) was started for five weeks. The animals in the CMS-R group were euthanized, while the CMS-S group continued to be subjected to stress and treatment with tPBM. The CMS-S group received treatment using tPBM, with each group having similar sucrose consumption averages through a pairing considering the sucrose consumption outcome. This led to the group’s division into three new subgroups based on the wavelengths of 600 nm (Red group), 840 nm (Infrared group), and a sham group. The animals in the non-stress control group were handled daily for the same period as those in the stress group. During the quarantine period, the animals were not handled, whereas during the baseline sucrose consumption period and the CVS protocol, the animals were handled, including the non-stressed (control) group.

### tPBM Treatment

Treatment with both wavelengths was transcranial. The animals were manually immobilized, and the light source was positioned in the frontal portion of the animal’s head (dorsal midline of the frontal region), aiming to stimulate the prefrontal cortex [27]. Table 1 describes the parameters used. All animals underwent trichotomy at the application site, as the hair makes it difficult for light to pass through. Treatment with transcranial photobiomodulation occurred once a day for five weeks. The use of 600 nm (red) and 840 nm (near-infrared) wavelengths in our study is justified by the broader therapeutic range of photobiomodulation (PBM) and its capacity to target cytochrome c oxidase (CCO) effectively within the red-to-NIR spectrum (600–1100 nm). Red light at 600 nm offers strong absorption by CCO, triggering mitochondrial activity and ATP production. Near-infrared light at 840 nm, on the other hand, penetrates deeper into tissue compared to shorter wavelengths, further enhancing the ability to modulate deeper cortical regions. While many studies focus on 630–810 nm, selecting 600 and 840 nm provides complementary wavelengths within the established range, optimizing the balance between tissue penetration and absorption by CCO [15, 17].

### Euthanasia and Collection of Biological Material

Twenty-four hours after the last sucrose consumption test, the animals were euthanized. Animal euthanasia followed the procedures and methods indicated by RN 37/2018 of the CONCEA Euthanasia Practice Guidelines and described in project nº 15–0353, previously approved. The method used was anesthetic with intranasal isoflurane overdose followed by decapitation. The brain was immediately removed and dissected, separating the region of the prefrontal cortex and the hippocampus. The truncal blood was drawn shortly after the decapitation. The tissues were frozen in liquid nitrogen for subsequent storage in an ultra-freezer (−80 °C) and biochemical evaluation.

### Measures of Lipid Damage and Antioxidant Defense on Peripheral Blood

#### Thiobarbituric Acid Reactive Substances (TBARS)

Lipid peroxidation was monitored by the formation of thiobarbituric acid reactive substances (TBARS) during an acid-heating reaction, according to Wills [28], with modifications [28]. Specifically, 400 µL of supernatant from each sample was combined with 600 µL of 15% trichloroacetic acid and 0,67% thiobarbituric acid. The mixture was heated at 100 °C for 15 min. After cooling to room temperature, the samples were centrifuged at 5200 xg for 30 min. The supernatants were isolated, and their absorbance was measured at 530 nm. Hydrolyzed malondialdehyde (MDA) was used as standard, and the results were expressed as nmol MDA/mL [28]. The tests were conducted at the Oxidative Stress and Antioxidants Laboratory at the University of Caxias do Sul/RS - Brazil.

#### Determination of Total Antioxidant Capacity Serum (TEAC)

The screening of antioxidant activity was performed through the sample’s ability to scavenge the radical ABTS+ [2,2-azino-bis (3-etilbenzotiazolin) −6-sulfonic acid]. The method follows the procedures described by Re et al., [29]. The ABTS+ solution is formed from the reaction of 7 mM ABTS with 2.45 mM potassium persulphate. This solution was kept in the dark at room temperature for 12–16 h before use. Then, the solution ABTS • + was diluted with 5mM phosphate buffer saline (PBS pH 7.4) until an absorbance of 0.700 ± 0.35 at 734 nm. Then, 1.0 mL of ABTS • + diluted solution was added to 10 µL of sample. The absorbances were read precisely 6 min after the initial mixture. A standard curve was used with Trolox solution for quantification, and the results were expressed in mM of equivalents of Trolox [29]. The tests were conducted at the Oxidative Stress and Antioxidants Laboratory at the University of Caxias do Sul/RS - Brazil.

### Central Measurements on Cerebral Tissue

#### Complex IV Activity in the Prefrontal Cortex

The activity of the mitochondrial chain complex IV, also known as cytochrome C oxidase (CCO), was assayed in the homogenized cerebral cortex [30]. The analyses were performed on all samples; however, due to the characteristics of the technique and the tissue under study, the samples were prepared in 3 pools per group. Complex IV activity was measured at 550 nm, and the positive control was potassium cyanide. Enzymatic activity was evaluated at 37 °C in a spectrophotometer (Shimadzu model UV-1700). The results were presented as nmol/min/mg protein. The tests were conducted at the Oxidative Stress and Antioxidants Laboratory at the University of Caxias do Sul/RS - Brazil.

#### Nitric Oxide Concentration in the Hippocampus

Nitric oxide (NO) bioavailability was assessed according to the Griess reaction based on the Green et al. [31] method [31]. Due to the characteristics of the technique and the tissue under study, the samples were prepared in 3 pools per group. The homogenized tissues were added to Griess reagent (1:1), mixed, and incubated in the dark for 10 min after reading at 550 nm by a microplate reader. Sodium nitroprusside was used as the standard. The results were expressed as nmol of nitrite/mg of protein. The tests were conducted at the Oxidative Stress and Antioxidants Laboratory at the University of Caxias do Sul/RS - Brazil. Given that neuroinflammation and metabolic dysfunction are closely interrelated in the pathophysiology of MDD, we intentionally included both aspects in our study. We believe that PBM’s potential therapeutic effects might operate through the modulation of these intertwined pathways, offering a more comprehensive understanding of its mechanisms of action.

### Statistical Analysis

We analyzed the data using the R platform with Dplyr and broom packages. The Shapiro-Wilk test for normality was used to verify whether each parameter followed a normal distribution before comparing groups. The analysis of variance (ANOVA) was applied to parametric data. At the same time, the Kruskal-Wallis was used to compare groups for non-parametric data, followed by Bonferroni´s pairwise grouping with a significance level of *p* < 0.05. The graphs were generated on *ggpubr* and *ggplot2*, also within the R platform.

## Results

### Sucrose Consumption

#### Baseline

**Fig. 1:**
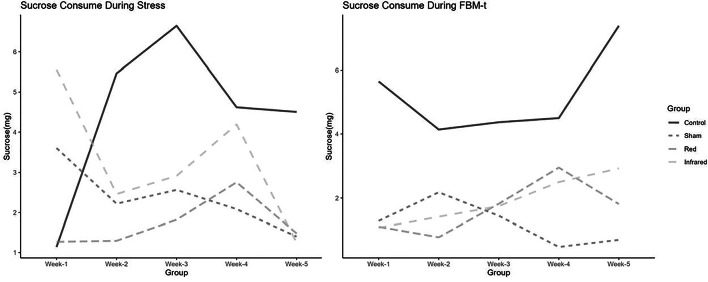
.

The groups did not differ significantly at the baseline before the chronic stress protocol (Kruskal-Wallis, *p* = 0.123). By the fifth week of protocol, the sham, red, and infrared groups showed lower sucrose consumption levels than the control group, without a statistical difference (Kruskal-Wallis chi-squared 7.5069; *p* = 0.0574). On the first day of the tPBM intervention, the control group consumed 5,396 g of sucrose, whereas the rats exposed to the stress protocol consumed 1,478 g (sham) (*p* = 0.00879). Indeed, the sucrose consumption was reduced by over 55% and 67% in the sham group and red/infrared groups, respectively, when compared to baseline measurements (Table 2; Fig. 1). It is important to note that over the five weeks of application of the stress protocol, sucrose consumption varied considerably between the groups. This variation, which is a normal aspect of the test, is greatly influenced by the environment, temperature, variations in the behavior of the rodents throughout the days, and the materials used for measurement [32].

**Table 2:** Sucrose consumption (g) during stress

	Baseline	Week 2	Week 3	Week 4	Week 5	Week 5/Baseline
	M (SD)					
Control	3.04 (3.38)	5.93 (4.53)	14.33 (23.98)	6.89 (5.77)	5.05 (4.14)	1.661
Infrared	5.67 (3.26)	4.42 (7.39)	10.61 (24.2)	4.30 (2.69)	1.81 (1.35)	0.319
Red	3.40 (4.01)	3.95 (7.01)	11.1 (28.88)	2.69(1.48)	1.83(1.32)	0.538
Sham	3.23 (2.29)	12.74 (31.07)	3.05 (2.67)	5.26(6.48)	1.80 (1.37)	0.557
Chi-squared	5.7761	3.3178	2.3691	3.5927	7.5069	
p-value	0.123	0.345	0.499	0.309	**0.0574**	

#### After Transcranial Photobiomodulation

**Fig. 2:**
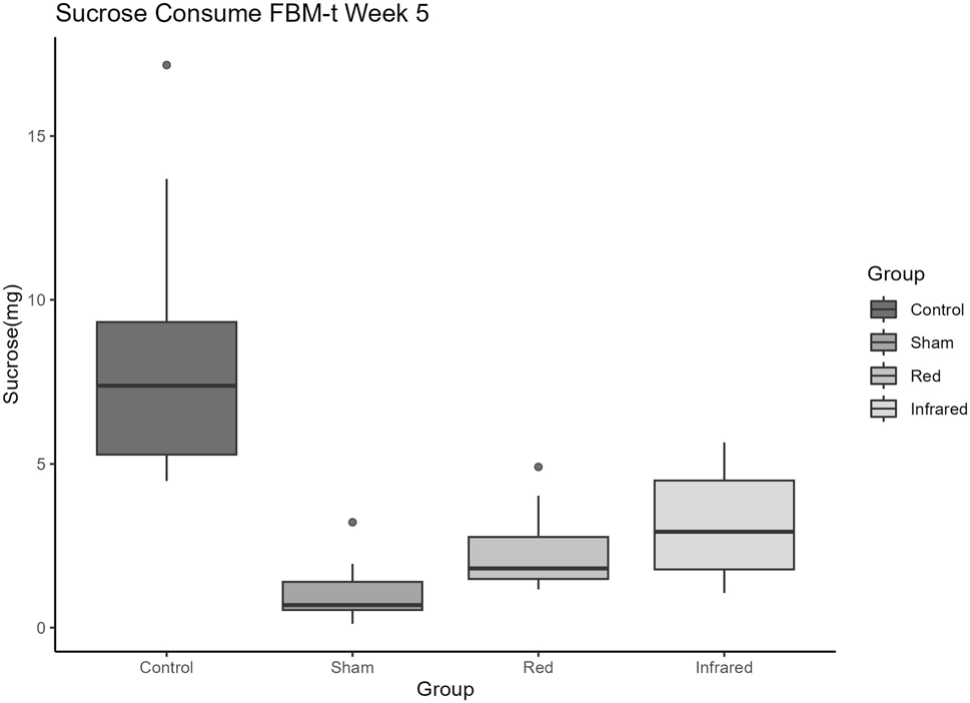
.

**Fig. 3:**
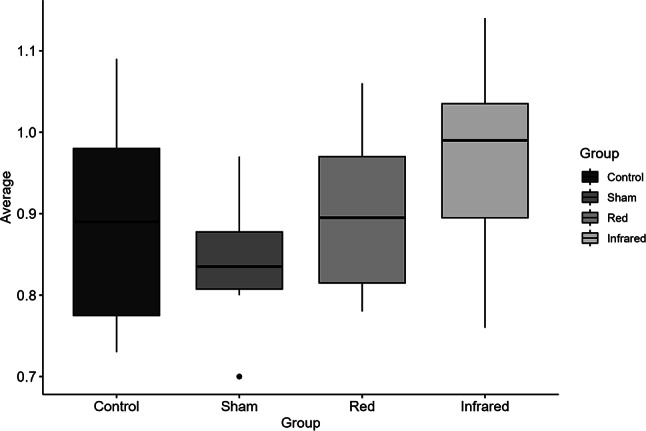
.

After five weeks of CMS, CMS-S rats received t-PBM for five weeks in addition to maintaining the CMS protocol for the same period. On the fifth week of treatment, both red and infrared groups exhibited increased sucrose consumption compared to the sham (Kruskal Wallis chi-squared = 26.131; *p* < 0.001). While the sham group remained in an anhedonic state, experiencing a 25% reduction in sucrose consumption, the red group more than doubled their consumption compared to the first day of the intervention. The infrared group recovered approximately 1,67 times their initial consumption level to the baseline. These data suggest that the tPBM using both infrared and red frequencies improved the anhedonic behavior in rats exposed to chronic stress protocol, as shown in Fig. 2. It is important to highlight that this difference concerning initial consumption in the treated groups (red and infrared) does not present a statistical difference compared to the sham group. Furthermore, after 5 weeks of treatment with photobiomodulation, the infrared group showed no statistical difference compared to the control group (Fig. 3).

### Peripheral Blood Biomarkers

#### Thiobarbituric Acid Reactive Substances (TBARS)

**Fig. 4:**
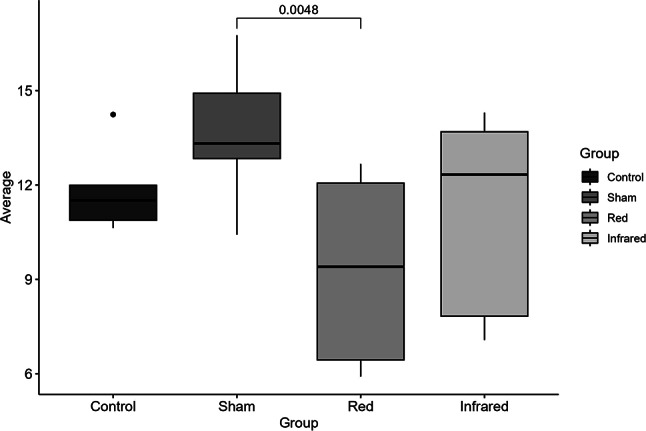
.

One-way ANOVA revealed statistical differences in TBARS levels among groups (F = 3.21; *p* = 0.0394). Post hoc analysis indicated that the red group showed lower TBARS levels (M = 9.50, SD = 2.87) than the sham group (M = 13.66, SD = 2.20) (*p* = 0.0048) and was similar to the control group (Fig. 4). There were no differences between infrared and other groups.

#### Trolox Equivalent Antioxidant Capacity (TEAC)

Although infrared and red groups presented higher TEAC levels than sham and control groups (Fig. 3), these effects did not reach statistical significance (*p* = 0.306).

### Central Biomarkers

#### Nitric Oxide (NO) on the Hippocampus

**Fig. 5:**
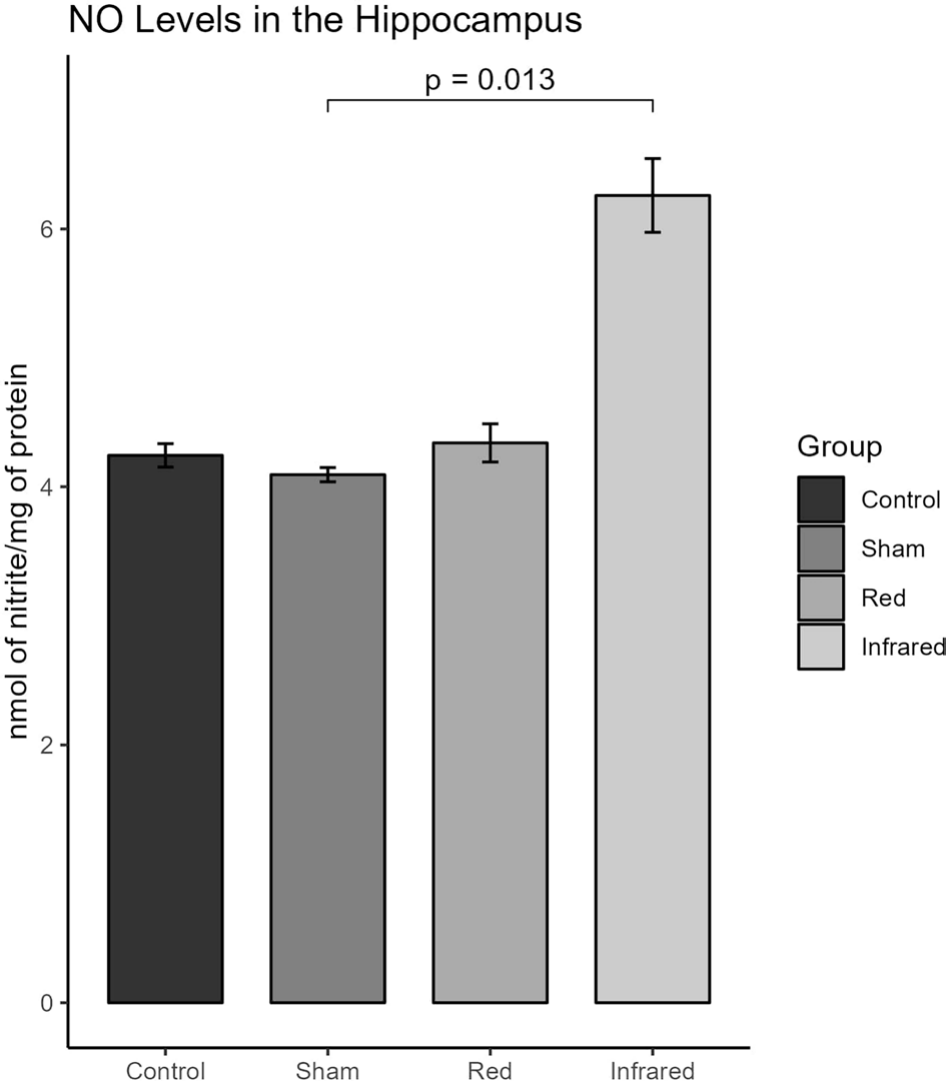
.

According to the Kruskal-Wallis test, there was a statistically significant difference in the hippocampal levels of NO among groups (Chi-squared = 9.6667; *p* = 0.02162). In particular, the NO levels within the hippocampus of rats treated with infrared significantly increased compared to the Sham group, although not with the other two groups (Mean = 107.83; SD = 6.48, Dunn Test *p* = 0.0134). Conversely, no differences were observed between the red and other groups, as shown in Fig. 5.

#### Mitochondrial Chain Complex IV Activity on the Prefrontal Cortex

**Fig. 6:**
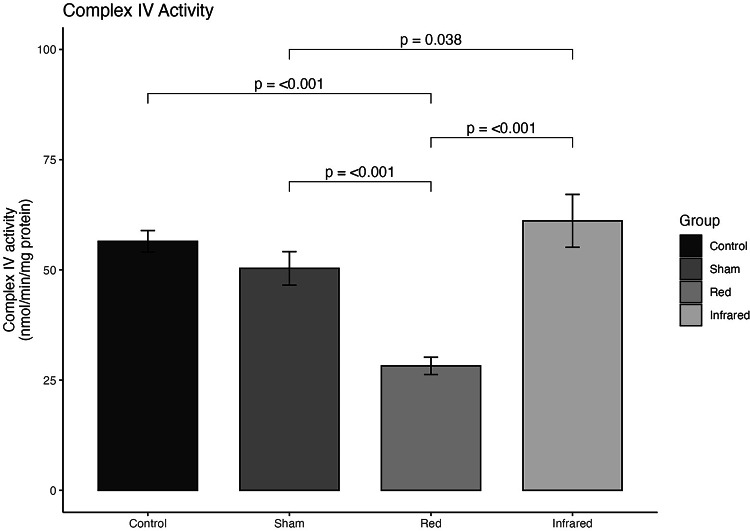
.

Complex IV activity differed significantly across experimental groups (*F*(3,8) = 42.26; *p* < 0.001; Fig. 6). The Sham group did not differ from Controls, indicating no effect of the sham manipulation. In contrast, red light exposure markedly reduced Complex IV activity compared with both Control and Sham groups (*p* < 0.001 for both). Conversely, infrared light significantly increased Complex IV activity compared with all other groups (vs. Control, *p* = 0.038; vs. Sham and Red, *p* < 0.001). These findings indicate that infrared irradiation exerts a stimulatory effect on mitochondrial activity, whereas red light exerts an inhibitory effect.

## Discussion

Our study showed that the CMS model efficiently reproduced anhedonic behavior showing a significant reduction of sucrose consumption in the groups exposed to this protocol. Furthermore, the tPBM red and infrared were able to reverse this depressive-like behavior, which suggests the promising antidepressant properties of this intervention. In the study by Xu et al. [33], it was also shown that photobiomodulation effectively improved depression-like behaviors in two mouse models of depression [33]. The CMS protocol is a well-established animal model for depressive-like behavior and is closely associated with anhedonic tendencies in rodents [34–36]. Anhedonia is considered a central symptom in depressive disorders and also serves as a marker for predicting the prognosis of treatment-resistant depression [37–39]. Indeed, anhedonia has been used as a primary outcome of distinct CMS [11]. The sucrose preference test, initially proposed by Willner [36] and posteriorly adapted by Papp [34] stands as a gold standard for assessing anhedonia in rats [34–36]. Our results showed the positive effects of tPBM in a valid animal model for depressive-like behavior, as observed in the study by Tanaka et al. [40].

Several theories exist to explain the complex causes of MDD, including the inflammatory theory [12]. Inflammation or the inflammatory response is a consequence of the activation of the immune system, typically appearing as a localized reaction. Its primary purpose is to rid the body of harmful stimuli or insults. Various immune cells and mechanisms are mobilized to safeguard the body’s internal equilibrium, known as homeostasis. In the brain, studies have shown that microglial activation leads to neuroinflammation in both animal models and clinically depressed patients [41]. Thus, it is important to note that the dysregulation of the immune processes can frequently play a role in developing diseases, including MDD [13]. In line with this, oxidative stress (OS) is a biological phenomenon arising from the disruption in the balance between the production of reactive oxygen species (ROS), commonly known as free radicals, and the body’s ability to neutralize them through antioxidative defenses [42]. The resultant impact of these ROS on cellular components can induce cellular dysfunction and trigger an inflammatory response, where inflammatory cells contribute to the generation of ROS and, consequently, OS [42, 43]. This connection reinforces the significant role of inflammation in mood disorders. Individuals with mood disturbances often display heightened levels of pro-inflammatory cytokines, oxidative stress products, chemokines, and soluble adhesion molecules in blood and cerebrospinal fluid [44]. Furthermore, systemic inflammation and OS can contribute to neuroinflammation, a well-established factor in developing neurodegenerative diseases and mood disorders [42].

The present study investigated the antioxidant and metabolic properties of tPBM using the TBARS, TEAC, NO, and mitochondrial complex on cortical structures and serum of rats exposed to CMS protocol. Regarding TBARS, red light reduced lipid peroxidation (lower TBARS) relative to sham, whereas infrared showed a similar trend without reaching significance. Total antioxidant capacity (TEAC) was unchanged across groups. Together, this suggests that red light attenuates oxidative damage primarily by reducing oxidant generation or lipid peroxidation rather than by boosting bulk antioxidant capacity. Infrared may have a smaller or more variable effect under these conditions. In this context, the study by Yang et al. (2011) demonstrated the ability of red light (632.8 nm) to suppress Aβ-induced ROS production and inflammatory response in rat primary astrocytes [45]. A study using a restraint stress model mice model showed that tPBM with infrared light (810 nm) and Coenzyme Q10 improved the antioxidant defense capacity of the brain [46]. In another study, Salehpour and colleagues showed that tPBM with infrared light reduced oxidative damage in the hippocampus, demonstrating the ability of 810 nm laser light to enhance the antioxidant defense system and maintain mitochondrial survival in sleep-deprived (SD) mice. Treatment with transcranial NIR laser in SD mice restored hippocampal total antioxidant capacity levels compared to those in the untreated SD group [27]. Banqueri et al., [47] investigated the effects of early life stress on cognitive flexibility and brain metabolism in rats, as well as the potential of photobiomodulation (PBM) as a treatment for these effects. As expected, the study found that early life stress led to long-term cognitive impairments, particularly in cognitive flexibility and an increase in brain energy metabolism, indicating disruptions in brain metabolism. Consistent with previous evidence highlighting the antioxidant and neuroprotective properties of tPBM, the present study demonstrates that red light stimulation significantly attenuated lipid peroxidation, as indicated by lower TBARS levels compared to sham stimulation. Although infrared light exposure produced a reduction in TBARS, this effect did not reach statistical significance under the current experimental parameters, which may reflect differences in wavelength-dependent tissue penetration, mitochondrial absorption spectra, or the need for optimized dosimetric conditions. In contrast to prior reports showing increases in total antioxidant capacity following tPBM—particularly with infrared wavelengths—no significant changes in TEAC were observed in either treatment group, suggesting that the observed reduction in lipid peroxidation with red light is more likely attributable to a direct decrease in oxidative damage rather than to enhanced overall antioxidant defenses. These findings contribute to the growing body of evidence supporting tPBM as a non-invasive strategy to modulate oxidative stress within the central nervous system, with potential implications for the prevention or mitigation of neurodegenerative and stress-related pathologies. Further research is necessary to elucidate the mechanisms underlying these effects and to optimize tPBM protocols for clinical applications targeting neurodegenerative diseases and stress-related disorders [47, 48].

Furthermore, our results demonstrated that tPMB, particularly infrared, improved CCO (mitochondrial chain complex IV) activity significantly more than sham, achieving levels of activity similar to those observed in the control non-stressed group. The mitochondrial complexes are sets of enzymatic proteins located in the inner membrane of mitochondria. These complexes are crucial in the respiratory chain, the central metabolic pathway for ATP generation. Four main complexes (I, II, III, and IV) comprise multiple protein subunits collaborating in electron transfer along the respiratory chain. Mitochondrial complexes are pivotal for cellular energy homeostasis, playing a fundamental role in the healthy functioning of cells and organisms [49]. Absorption of photon energy by the CCO is proposed to be a central mechanism for the PBM effect [50]. For instance, laser treatment using both red and infrared (660 and 810 nm) has shown decreased reactive oxygen species (ROS) production and increased cytochrome c oxidase (CCO) compared to sham in the D-Galactose-induced aging model [51]. Another research identified changes in CCO activity within cortical and subcortical regions after tPBM with an infrared laser administration (1,064 nm) [52]. The study by Wade et al. [52] reports that the most significant shifts in CCO activity occurred one day after tPBM with infrared light application in the infralimbic prefrontal cortex, with effects persisting elevated for 2 to 4 weeks post-tPBM [52]. Moreover, significant differences in CCO activity between the 2-week and sham groups were observed in various brain regions, including the molecular layer of the hippocampus, the CA3 region of the hippocampus, the lateral septum, and the nucleus accumbens. Interregional correlation analysis indicated enhanced functional connectivity between cortical and subcortical areas post-tPBM, which endured for four weeks post-stimulation. The temporal pattern of changes in CCO activity and functional coupling suggests the occurrence of distinct forms of neuroenergetic plasticity at different time intervals post-tPBM, contingent upon the brain region and its cortical depth. In this context, evidence has shown that in vivo oxidation of CCO is a direct photonic action of administration of tPBM to the human prefrontal cortex. Upon penetrating the skull, the tPBM is absorbed by CCO, leading to a direct and non-thermal photonic oxidation of CCO [41]. Wang et al. [53] demonstrated that tPBM-NIR positively regulates oxidized cytochrome c oxidase (CCO) in the human brain, a finding corroborated by Pruitt et al. [54], who also observed an increase in the concentration of oxidized CCO [53, 54]. Saucedo et al. [55] advocate using tPBM to enhance cerebral oxygenation and alleviate age-related declines in mitochondrial respiration [55]. They found significant elevations in ATP biosynthesis and the expression and activity levels of mitochondrial complex IV in the prefrontal cortex (PFC) following PBM. Additionally, Zhang et al. [56] and Huang et al., [57] have suggested the protective effects of PBM could be attributed to the increased ATP production and selective modulation of pro-inflammatory mediators [56, 57]. Collectively, these studies underscore the impact of infrared tPBM on enhancing the bioenergetic capacity of the brain. In agreement with this evidence, our findings demonstrate that tPBM, particularly at infrared wavelengths, effectively restores mitochondrial complex IV activity to levels comparable to non-stressed controls, aligning with previous evidence that CCO is a primary photoreceptor for PBM and that its photonic activation enhances mitochondrial bioenergetics, ATP synthesis, and neuroenergetic plasticity, thereby supporting the role of infrared tPBM as a potent modulator of cerebral energy metabolism.

In our study, the t-PBM, particularly with NIR, increased nitric oxide (NO) in the hippocampus of treated animals. This may be correlated with the increased activity of CCO. This correlation arises from the fact that CCO can be inhibited by NO. PBM can dissociate NO from the CCO, leading to elevated mitochondrial membrane potential, increased oxygen consumption, enhanced glucose metabolism, and more outstanding ATP production by the mitochondria [19]. It is important to highlight that the increase in NO concentration observed in the tPBM near-infrared group may be related to the increase in complex IV activity, resulting in its flow into the cytosol [58]. When released from mitochondrial regulatory sites, NO becomes available in the mitochondria and the cytosol, which may explain the higher NO levels observed in this group. Released NO can also act as a vasodilator, increasing blood flow and improving metabolism in the t-PBM irradiated area, also contributing to the antidepressant effect [50]. It is important to mention that we did not assess nitric oxide in the prefrontal cortex due to technical constraints. The prefrontal cortex of rats is a very small structure, making it unfeasible to measure both complex IV activity and nitric oxide levels simultaneously due to sample size limitations. Since the hippocampus is a fundamental structure for improving outcomes related to anhedonic behavior and demonstrates a close relationship with the prefrontal cortex, we chose this structure to infer the relationship between the stages of complex IV activity and the interaction of this complex with NO. While the proposed mechanism—that CCO dissociates NO from CCO, leading to increased CCO activity and, subsequently, higher cytosolic NO levels is a plausible hypothesis, we believe that the fact that these measurements were made in different brain regions is a limitation of the study. The effects of tPBM may not be uniform across the brain; instead, they may be region-specific or even part of a complex cascade where a change in one area influences another.

Other limitations of this study should be considered such as the appropriate dosage of tPMB for bio-stimulation induction. This is because of factors such as wavelength, output power, continuous or pulsed emission, power density, irradiation time, dose in J/cm2, total delivered energy, application technique, and intervals between sessions [59]. Additionally, the three minutes of immobilization utilized in this experiment for tPBM application may have induced physical stress in the rats, potentially influencing the antidepressant effects of phototherapy. Furthermore, the CMS protocol has its own limitations, such as sex differences and interindividual vulnerability to stress, which are yet unexplored in this model [60]. We recommend that future investigations include animals of both sexes to determine whether the observed effects are consistent across sexes. Although this limitation does not compromise the validity of the present study’s procedures, it underscores the need for additional research to elucidate the applicability of tPBM in a broader population. Furthermore, despite the strong theoretical framework provided by the inflammatory hypothesis of depression, the present study did not directly assess inflammatory markers. Future studies should therefore examine microglial morphology or quantify cytokine levels to establish a more definitive link between tPBM and neuroinflammatory processes.

## Conclusion

In summary, our findings suggest that tPBM holds promise as an effective antidepressant strategy. The improvement in sucrose consumption, the increase in Complex IV activity in the prefrontal cortex, and the increase in NO concentration in the hippocampus, associated with a reduction in damage to membrane lipids peripherally, may be indicators of the beneficial effects of this therapy. For most of the outcomes assessed in this study, the NIR wavelength was superior or similar to red light, suggesting NIR should be preferred for future studies.

## Data Availability

The de-identified database used in the current study is available from the corresponding author upon reasonable request.

## Abbreviations

MDD: Major depressive disorder
TRD: Treatment-resistant depression
CMS: Chronic mild stress
ROS: Reactive oxygen species
PBM: Photobiomodulation
tPBM: Transcranial photobiomodulation
CCO: Cytochrome C oxidase
ATP: Adenosine triphosphate
NO: Nitric oxide
TBARS: Thiobarbituric acid reactive substances
TEAC: Total antioxidant capacity serum

## References

[1] Dadkhah M, Jafarzadehgharehziaaddin M, Molaei S, Akbari M, Gholizadeh N, Fathi F (2023) Major depressive disorder: biomarkers and biosensors. Clin Chim Acta 547:117437. 10.1016/j.cca.2023.117437 37315724 10.1016/j.cca.2023.117437

[2] Marx W, Penninx BWJH, Solmi M, Furukawa TA, Firth J, Carvalho AF, Berk M (2023) Major depressive disorder. Nat Rev Dis Primers 9:1–21. 10.1038/s41572-023-00454-1 37620370 10.1038/s41572-023-00454-1

[3] Malhi GS, Mann JJ (2018) Depression. Lancet 392:2299–2312. 10.1016/S0140-6736(18)31948-2 30396512 10.1016/S0140-6736(18)31948-2

[4] Arnaud AM, Brister TS, Duckworth K, Foxworth P, Fulwider T, Suthoff ED, Werneburg B, Aleksanderek I, Reinhart ML (2022) Impact of Major Depressive Disorder on comorbidities: a systematic literature review. J Clin Psychiatry 83:43390. 10.4088/JCP.21r14328 10.4088/JCP.21r14328 36264099

[5] Rajan S, McKee M, Rangarajan S, Bangdiwala S, Rosengren A, Gupta R, Kutty VR, Wielgosz A, Lear S, AlHabib KF, Co HU, Lopez-Jaramillo P, Avezum A, Seron P, Oguz A, Kruger IM, Diaz R, Nafiza M-N, Chifamba J, Yeates K, Kelishadi R, Sharief WM, Szuba A, Khatib R, Rahman O, Iqbal R, Bo H, Yibing Z, Wei L, Yusuf S (2020) Association of symptoms of depression with cardiovascular disease and mortality in low-, middle-, and high-income countries. JAMA Psychiatr 77:1052–1063. 10.1001/jamapsychiatry.2020.1351 10.1001/jamapsychiatry.2020.1351 PMC7287938 32520341

[6] Silva AR, Sgnaolin V, Nogueira EL, Loureiro F, Engroff P, Gomes I (2017) Doenças crônicas não transmissíveis e fatores sociodemográficos associados a Sintomas de depressão Em Idosos. J Bras Psiquiatr 66:45–51. 10.1590/0047-2085000000149

[7] Su P, Yan S, Yang J, Tong J, Samsom J, You F, Li Y, Chen Q, Jiang A, Zhai D, Chen J, Sun Z, Zhou J, Liu M, Lee FJS, Xu Z-QD, Wang X, Vasdev N, Wong AHC, Liu F (2023) Serum amyloid P component (SAP) modulates antidepressant effects through promoting membrane insertion of the serotonin transporter. Neuropsychopharmacology 48:508–517. 10.1038/s41386-022-01449-4 36076020 10.1038/s41386-022-01449-4 PMC9852251

[8] McAllister-Williams RH, Arango C, Blier P, Demyttenaere K, Falkai P, Gorwood P, Hopwood M, Javed A, Kasper S, Malhi GS, Soares JC, Vieta E, Young AH, Papadopoulos A, Rush AJ (2020) The identification, assessment and management of difficult-to-treat depression: an international consensus statement. J Affect Disord 267:264–282. 10.1016/j.jad.2020.02.023 32217227 10.1016/j.jad.2020.02.023

[9] Amasi-Hartoonian N, Pariante CM, Cattaneo A, Sforzini L (2022) Understanding treatment-resistant depression using omics techniques: a systematic review. J Affect Disord 318:423–455. 10.1016/j.jad.2022.09.011 36103934 10.1016/j.jad.2022.09.011

[10] Strekalova T, Liu Y, Kiselev D, Khairuddin S, Chiu JLY, Lam J, Chan YS, Pavlov D, Proshin A, Lesch KP, Anthony DC (2022) Chronic mild stress paradigm as a rat model of depression: facts, artifacts, and future perspectives. Psychopharmacology 239(3):663–693 35072761 10.1007/s00213-021-05982-w PMC8785013

[11] Antoniuk S, Bijata M, Ponimaskin E, Wlodarczyk J (2019) Chronic unpredictable mild stress for modeling depression in rodents: meta-analysis of model reliability. Neurosci Biobehav Rev 99:101–116. 10.1016/j.neubiorev.2018.12.002 30529362 10.1016/j.neubiorev.2018.12.002

[12] Gałecki P, Talarowska M (2018) Inflammatory theory of depression. Psychiatr Pol 52:437–447. 10.12740/PP/76863 30218560 10.12740/PP/76863

[13] Beurel E, Toups M, Nemeroff CB (2020) The bidirectional relationship of depression and inflammation: double trouble. Neuron 107:234. 10.1016/j.neuron.2020.06.002 32553197 10.1016/j.neuron.2020.06.002 PMC7381373

[14] Caldieraro MA, Cassano P (2019) Transcranial and systemic photobiomodulation for major depressive disorder: a systematic review of efficacy, tolerability and biological mechanisms. J Affect Disord 243:262–273. 10.1016/j.jad.2018.09.048 30248638 10.1016/j.jad.2018.09.048

[15] Salehpour F, Gholipour-Khalili S, Farajdokht F, Kamari F, Walski T, Hamblin MR, DiDuro JO, Cassano P (2020) Therapeutic potential of intranasal photobiomodulation therapy for neurological and neuropsychiatric disorders: a narrative review. Rev Neurosci 31:269–286. 10.1515/revneuro-2019-0063 31812948 10.1515/revneuro-2019-0063 PMC7138738

[16] Cassano P, Cusin C, Mischoulon D, Hamblin MR, De Taboada L, Pisoni A, Chang T, Yeung A, Ionescu DF, Petrie SR, Nierenberg AA, Fava M, Iosifescu DV (2015) Near-infrared transcranial radiation for major depressive disorder: proof of concept study. Psychiatr J 2015:352979. 10.1155/2015/352979 10.1155/2015/352979 PMC4556873 26356811

[17] Montazeri K, Farhadi M, Fekrazad R, Chaibakhsh S, Mahmoudian S (2022) Photobiomodulation therapy in mood disorders: a systematic review. Lasers Med Sci 37:3343–3351. 10.1007/s10103-022-03641-w 36404359 10.1007/s10103-022-03641-w

[18] Cardoso F, de Souza Oliveira Tavares C, Araujo BHS, Mansur F, Lopes-Martins RÁB, da Gomes Silva S (2022) Improved spatial memory and neuroinflammatory profile changes in aged rats submitted to photobiomodulation therapy. Cell Mol Neurobiol 42:1875–1886. 10.1007/s10571-021-01069-4 33704604 10.1007/s10571-021-01069-4 PMC11421705

[19] Hamblin MR (2017) Mechanisms and applications of the anti-inflammatory effects of photobiomodulation. AIMS Biophys 4:337–361. 10.3934/biophy.2017.3.337 28748217 10.3934/biophy.2017.3.337 PMC5523874

[20] Wang Q-S, Tian J-S, Cui Y-L, Gao S (2014) Genipin is active via modulating monoaminergic transmission and levels of brain-derived neurotrophic factor (BDNF) in rat model of depression. Neuroscience 275:365–373. 10.1016/j.neuroscience.2014.06.032 24972301 10.1016/j.neuroscience.2014.06.032

[21] Golden SA, Covington HE, Berton O, Russo SJ (2011) A standardized protocol for repeated social defeat stress in mice. Nat Protoc 6:1183–1191. 10.1038/nprot.2011.361 21799487 10.1038/nprot.2011.361 PMC3220278

[22] Nieto-Gonzalez JL, Holm MM, Vardya I, Christensen T, Wiborg O, Jensen K (2015) Presynaptic plasticity as a hallmark of rat stress susceptibility and antidepressant response. PLoS One 10:e0119993. 10.1371/journal.pone.0119993 25742132 10.1371/journal.pone.0119993 PMC4350919

[23] Papp M (2012) Models of affective illness: chronic mild stress in the rat. Curr Protoc Pharmacol. 10.1002/0471141755.ph0509s57 22684722 10.1002/0471141755.ph0509s57

[24] Papp M, Willner P (2023) Models of affective illness: chronic mild stress in the rat. Curr Protoc 3:e712. 10.1002/cpz1.712 36892313 10.1002/cpz1.712

[25] Papp M, Gruca P, Lason M, Tota-Glowczyk K, Niemczyk M, Litwa E, Willner P (2018) Rapid antidepressant effects of deep brain stimulation of the pre-frontal cortex in an animal model of treatment-resistant depression. J Psychopharmacol 32:1133–1140. 10.1177/0269881118791737 30182787 10.1177/0269881118791737

[26] Willner P, Gruca P, Lason M, Tota-Glowczyk K, Litwa E, Niemczyk M, Papp M (2019) Validation of chronic mild stress in the Wistar-Kyoto rat as an animal model of treatment-resistant depression. Behav Pharmacol 30:239–250. 10.1097/FBP.0000000000000431 30204592 10.1097/FBP.0000000000000431

[27] Salehpour F, Farajdokht F, Erfani M, Sadigh-Eteghad S, Shotorbani SS, Hamblin MR, Karimi P, Rasta SH, Mahmoudi J (2018) Transcranial near-infrared photobiomodulation attenuates memory impairment and hippocampal oxidative stress in sleep-deprived mice. Brain Res 1682:36–43. 10.1016/j.brainres.2017.12.040 29307593 10.1016/j.brainres.2017.12.040 PMC5801165

[28] Wills ED (1966) Mechanisms of lipid peroxide formation in animal tissues. Biochem J 99:667–676. 10.1042/bj0990667 5964963 10.1042/bj0990667 PMC1265056

[29] Re R, Pellegrini N, Proteggente A, Pannala A, Yang M, Rice-Evans C (1999) Antioxidant activity applying an improved ABTS radical cation decolorization assay. Free Radic Biol Med 26:1231–1237. 10.1016/s0891-5849(98)00315-3 10381194 10.1016/s0891-5849(98)00315-3

[30] Spinazzi M, Casarin A, Pertegato V, Salviati L, Angelini C (2012) Assessment of mitochondrial respiratory chain enzymatic activities on tissues and cultured cells. Nat Protoc 7:1235–1246. 10.1038/nprot.2012.058 22653162 10.1038/nprot.2012.058

[31] Green LC, Wagner DA, Glogowski J, Skipper PL, Wishnok JS, Tannenbaum SR (1982) Analysis of nitrate, nitrite, and [15 N]nitrate in biological fluids. Anal Biochem 126:131–138. 10.1016/0003-2697(82)90118-x 7181105 10.1016/0003-2697(82)90118-x

[32] Liu MY, Yin CY, Zhu LJ, Zhu XH, Xu C, Luo CX, Chen H, Zhu DY, Zhou QG (2018) Sucrose preference test for measurement of stress-induced anhedonia in mice. Nat Protoc 13(7):1686–1698. 10.1038/s41596-018-0011-z 29988104 10.1038/s41596-018-0011-z

[33] Xu Z, Guo X, Yang Y, Tucker D, Lu Y, Xin N, Zhang G, Yang L, Li J, Du X, Zhang Q, Xu X (2017) Low-level laser irradiation improves depression-like behaviors in mice. Mol Neurobiol 54:4551–4559. 10.1007/s12035-016-9983-2 27379735 10.1007/s12035-016-9983-2 PMC5215986

[34] Papp M, Klimek V, Willner P (1994) Parallel changes in dopamine D2 receptor binding in limbic forebrain associated with chronic mild stress-induced anhedonia and its reversal by imipramine. Psychopharmacology 115:441–446. 10.1007/BF02245566 7871087 10.1007/BF02245566

[35] Willner P (2017) The chronic mild stress (CMS) model of depression: history, evaluation and usage. Neurobiol Stress 6:78–93. 10.1016/j.ynstr.2016.08.002 28229111 10.1016/j.ynstr.2016.08.002 PMC5314424

[36] Willner P, Towell A, Sampson D, Sophokleous S, Muscat R (1987) Reduction of sucrose preference by chronic unpredictable mild stress, and its restoration by a tricyclic antidepressant. Psychopharmacology (Berl.) 93:358–364. 10.1007/BF00187257 3124165 10.1007/BF00187257

[37] Coccurello R (2019) Anhedonia in depression symptomatology: appetite dysregulation and defective brain reward processing. Behav Brain Res 372:112041. 10.1016/j.bbr.2019.112041 31220485 10.1016/j.bbr.2019.112041

[38] Scheggi S, De Montis MG, Gambarana C (2018) Making sense of rodent models of anhedonia. Int J Neuropsychopharmacol 21:1049–1065. 10.1093/ijnp/pyy083 30239762 10.1093/ijnp/pyy083 PMC6209858

[39] Stanton CH, Holmes AJ, Chang SWC, Joormann J (2019) From stress to anhedonia: molecular processes through functional circuits. Trends Neurosci 42:23–42. 10.1016/j.tins.2018.09.008 30327143 10.1016/j.tins.2018.09.008 PMC6344037

[40] Tanaka Y, Akiyoshi J, Kawahara Y, Ishitobi Y, Hatano K, Hoaki N, Mori A, Goto S, Tsuru J, Matsushita H, Hanada H, Kodama K, Isogawa K, Kitamura H, Fujikura Y (2011) Infrared radiation has potential antidepressant and anxiolytic effects in animal model of depression and anxiety. Brain Stimulat 4:71–76. 10.1016/j.brs.2010.04.001 10.1016/j.brs.2010.04.001 21511206

[41] Wang H, He Y, Sun Z, Ren S, Liu M, Wang G, Yang J (2022) Microglia in depression: an overview of microglia in the pathogenesis and treatment of depression. J Neuroinflammation. 10.1186/s12974-022-02492-0 35668399 10.1186/s12974-022-02492-0 PMC9168645

[42] Teleanu DM, Niculescu AG, Lungu II, Radu CI, Vladâcenco O, Roza E, Costăchescu B, Grumezescu AM, Teleanu RI (2022) An overview of oxidative stress, neuroinflammation and neurodegenerative diseases. Int J Mol Sci. 10.3390/ijms23115938 35682615 10.3390/ijms23115938 PMC9180653

[43] Lima D, Cyrino LAR, Ferreira GK, Magro DDD, Calegari CR, Cabral H, Cavichioli N, Ramos SA, Ullmann OM, Mayer Y, Pscheidt LC, Schramm MA, Tomasi MC, Stammerjohann FLS, Delmonego L, Packer MH, Fiamoncini H (2022) Neuroinflammation and neuroprogression produced by oxidative stress in euthymic bipolar patients with different onset disease times. Sci Rep. 10.1038/s41598-022-21170-y 36202963 10.1038/s41598-022-21170-y PMC9537234

[44] Wei Y, Wang T, Li G, Feng J, Deng L, Xu H, Yin L, Ma J, Chen D, Chen J (2022) Investigation of systemic immune-inflammation index, neutrophil/high-density lipoprotein ratio, lymphocyte/high-density lipoprotein ratio, and monocyte/high-density lipoprotein ratio as indicators of inflammation in patients with schizophrenia and bipolar disorder. Front Psychiatry. 10.3389/fpsyt.2022.941728 35958647 10.3389/fpsyt.2022.941728 PMC9360542

[45] Yang X, Askarova S, Sheng W, Chen JK, Sun AY, Sun GY, Yao G, Lee JC-M (2010) Low energy laser light (632.8 nm) suppresses amyloid-β peptide-induced oxidative and inflammatory responses in astrocytes. Neuroscience 171:859–868. 10.1016/j.neuroscience.2010.09.025 20884337 10.1016/j.neuroscience.2010.09.025 PMC2987533

[46] Salehpour F, Cassano P, Rouhi N, Hamblin MR, De Taboada L, Farajdokht F, Mahmoudi J (2019) Penetration profiles of visible and near-infrared lasers and light-emitting diode light through the head tissues in animal and human species: a review of literature. Photobiomodulation Photomed Laser Surg 37:581–595. 10.1089/photob.2019.4676 10.1089/photob.2019.4676 31553265

[47] Banqueri M, Martínez JA, Prieto MJ, Cid-Duarte S, Méndez M, Arias JL (2019) Photobiomodulation rescues cognitive flexibility in early stressed subjects. Brain Res 1720:146300. 10.1016/j.brainres.2019.146300 31226326 10.1016/j.brainres.2019.146300

[48] Gutiérrez-Menéndez A, Cid-Duarte S, Banqueri M, Martínez JA, Méndez M, Arias JL (2021) Photobiomodulation effects on active brain networks during a spatial memory task. Physiol Behav 230:113291. 10.1016/j.physbeh.2020.113291 33338484 10.1016/j.physbeh.2020.113291

[49] Sousa JS, D’Imprima E, Vonck J (2018) Mitochondrial respiratory chain complexes. Subcell Biochem 87:167–227. 10.1007/978-981-10-7757-9_7 29464561 10.1007/978-981-10-7757-9_7

[50] Hamblin MR (2016) Shining light on the head: photobiomodulation for brain disorders. BBA Clin 6:113–124. 10.1016/j.bbacli.2016.09.002 27752476 10.1016/j.bbacli.2016.09.002 PMC5066074

[51] Salehpour F, Ahmadian N, Rasta SH, Farhoudi M, Karimi P, Sadigh-Eteghad S (2017) Transcranial low-level laser therapy improves brain mitochondrial function and cognitive impairment in D-galactose-induced aging mice. Neurobiol Aging 58:140–150. 10.1016/j.neurobiolaging.2017.06.025 28735143 10.1016/j.neurobiolaging.2017.06.025

[52] Wade ZS, Barrett DW, Davis RE, Nguyen A, Venkat S, Gonzalez-Lima F (2023) Histochemical mapping of the duration of action of photobiomodulation on cytochrome c oxidase in the rat brain. Front Neurosci 17:1243527. 10.3389/fnins.2023.1243527 37700747 10.3389/fnins.2023.1243527 PMC10493319

[53] Wang X, Tian F, Reddy DD, Nalawade SS, Barrett DW, Gonzalez-Lima F, Liu H (2017) Up-regulation of cerebral cytochrome-c-oxidase and hemodynamics by transcranial infrared laser stimulation: a broadband near-infrared spectroscopy study. J Cereb Blood Flow Metab 37:3789–3802. 10.1177/0271678X17691783 28178891 10.1177/0271678X17691783 PMC5718323

[54] Pruitt T, Wang X, Wu A, Kallioniemi E, Husain MM, Liu H (2020) Transcranial photobiomodulation (tPBM) with 1,064-nm laser to improve cerebral metabolism of the human brain in vivo. Lasers Surg Med 52:807–813. 10.1002/lsm.23232 32173886 10.1002/lsm.23232 PMC7492377

[55] Saucedo CL, Courtois EC, Wade ZS, Kelley MN, Kheradbin N, Barrett DW, Gonzalez-Lima F (2021) Transcranial laser stimulation: mitochondrial and cerebrovascular effects in younger and older healthy adults. Brain Stimulat 14:440–449. 10.1016/j.brs.2021.02.011 10.1016/j.brs.2021.02.011 33636401

[56] Zhang Q, Zhou C, Hamblin MR, Wu MX (2014) Low-level laser therapy effectively prevents secondary brain injury induced by immediate early responsive gene X-1 deficiency. J Cereb Blood Flow Metab 34:1391–1401. 10.1038/jcbfm.2014.95 24849666 10.1038/jcbfm.2014.95 PMC4126101

[57] Huang Y-Y, Nagata K, Tedford CE, McCarthy T, Hamblin MR (2013) Low-level laser therapy (LLLT) reduces oxidative stress in primary cortical neurons in vitro. J Biophotonics 6:829–838. 10.1002/jbio.201200157 23281261 10.1002/jbio.201200157 PMC3651776

[58] Ghasemi M, Mayasi Y, Hannoun A, Eslami SM, Carandang R (2018) Nitric oxide and mitochondrial function. Neurol Dis Neurosci 376:48–71. 10.1016/j.neuroscience.2018.02.017 10.1016/j.neuroscience.2018.02.017 29462705

[59] Dompe C, Moncrieff L, Matys J, Grzech-Leśniak K, Kocherova I, Bryja A, Bruska M, Dominiak M, Mozdziak P, Skiba THI, Shibli JA, Angelova Volponi A, Kempisty B, Dyszkiewicz-Konwińska M (2020) Photobiomodulation-underlying mechanism and clinical applications. J Clin Med 9:1724. 10.3390/jcm9061724 32503238 10.3390/jcm9061724 PMC7356229

[60] Loizeau V, Durieux L, Mendoza J, Wiborg O, Barbelivien A, Lecourtier L (2024) Behavioural characteristics and sex differences of a treatment-resistant depression model: chronic mild stress in the Wistar-Kyoto rat. Behav Brain Res 457:114712. 10.1016/j.bbr.2023.114712 37838247 10.1016/j.bbr.2023.114712

